# Endometriosis in Adolescents: Diagnostics, Clinical and Laparoscopic Features

**DOI:** 10.3390/jcm12041678

**Published:** 2023-02-20

**Authors:** Elena P. Khashchenko, Elena V. Uvarova, Timur Kh. Fatkhudinov, Vladimir D. Chuprynin, Aleksandra V. Asaturova, Elena A. Kulabukhova, Mikhail Yu. Vysokikh, Elvina Z. Allakhverdieva, Maria N. Alekseeva, Leila V. Adamyan, Gennady T. Sukhikh

**Affiliations:** 1 FSBI “National Medical Research Center for Obstetrics, Gynecology and Perinatology Named after Academician V.I. Kulakov” Ministry of Healthcare of the Russian Federation, 4, Oparina Street, 117997 Moscow, Russia; 2 Department for Obstetrics, Gynecology, Perinatology and Reproduction, Sechenov First Moscow State Medical University, Trubetskaya Str. 8, Bld. 2, 119991 Moscow, Russia; 3 Department of Histology, Cytology and Embryology, Peoples’ Friendship University of Russia (RUDN University), 117997 Moscow, Russia; 4 A.N. Belozersky Research Institute of Physico-Chemical Biology MSU, Leninskye Gory, House 1, Building 40, 119992 Moscow, Russia; 5 Faculty of Fundamental Medicine, Moscow State University Named after M.V. Lomonosov, 119991 Moscow, Russia

**Keywords:** peritoneal endometriosis, dysmenorrhea, pelvic pain, magnetic resonance imaging, histological examination, laparoscopy, CA-125, adolescents

## Abstract

Background: The early diagnosis of endometriosis in adolescents is not developed. Objective: We aim to conduct clinical, imaging, laparoscopic and histological analyses of peritoneal endometriosis (PE) in adolescents in order to improve early diagnosis. Methods: In total, 134 girls (from menarche to 17 years old) were included in a case–control study: 90 with laparoscopically (LS) confirmed PE, 44 healthy controls underwent full examination and LS was analyzed in the PE group. Results: Patients with PE were characterized with heredity for endometriosis, persistent dysmenorrhea, decreased daily activity, gastrointestinal symptoms, higher LH, estradiol, prolactin and Ca-125 (<0.05 for each). Ultrasound detected PE in 3.3% and MRI in 78.9%. The most essential MRI signs are as follows: hypointense foci, the heterogeneity of the pelvic tissue (paraovarian, parametrial and rectouterine pouch) and sacro-uterine ligaments lesions (<0.05 for each). Adolescents with PE mostly exhibit initial rASRM stages. Red implants correlated with the rASRM score, and sheer implants correlated with pain (VAS score) (<0.05). In 32.2%, foci consisted of fibrous, adipose and muscle tissue; black lesions were more likely to be histologically verified (0.001). Conclusion: Adolescents exhibit mostly initial PE stages, which are associated with greater pain. Persistent dysmenorrhea and detected MRI parameters predict the laparoscopic confirmation of initial PE in adolescents in 84.3% (OR 15.4; <0.01), justifying the early surgical diagnostics and shortening the time delay and suffering of the young patients.

## 1. Introduction

According to different authors, endometriosis occurs in 10–16% of women of early reproductive age and frequently manifests with chronic pelvic pain and dysmenorrhea in adolescence [1,2]. The exact prevalence of endometriosis in adolescents is underestimated, but at least in two-thirds of girls with chronic pelvic pain resistant to non-steroidal anti-inflammatory drugs (NSAIDs) or combined oral contraceptives (COCs), diagnostic laparoscopy confirms endometriosis [3,4]. The prevalence of endometriosis among adolescents was assessed in a systematic review by Janssen E. et al. based on 15 studies conducted between 1980 and 2011, which included 880 adolescent girls with dysmenorrhea or pelvic pain; endometriosis was laparoscopically confirmed in 62% of cases [3]. Currently, the severe forms of endometriosis occur up to 13–23% in young patients, causing a dramatically reduced quality of life while increasing the risks of repeated surgical interventions and future infertility [5,6,7]. 

Due to the growing recognition that endometriosis often manifests itself and progresses from adolescence, its early diagnosis is especially important [5,8,9,10,11]. On average, it takes 5–10 years from the symptoms’ onset to diagnosis; at the same time, adolescents expect help three times longer than adult women (6.0 ± 0.2 vs. 2.0 ± 0.3 years, *p* < 0.0001) [1,4]. The difficulty in diagnosing endometriosis in adolescents is determined by a non-specific clinical picture, the lack of noninvasive tests and the difficulty in detecting the initial forms of the disease using imaging modalities [12]. Ballweg M. et al., after analyzing the data of patients with endometriosis at an early reproductive age, concluded that the delay in diagnosis was largely due to the fact that doctors were not ready to diagnose endometriosis at an early age; moreover, before a correct diagnosis was established, patients were examined by four [13] and even up to seven general practitioners according to Eisenberg V. et al.’s data [4]. The presence of inflammatory, neuroendocrine and chronic comorbid conditions, which are also common in adolescents, makes the diagnosis even more difficult [14].

In adult women, endometriosis most typically manifests with cyclic pelvic pains, whereas in adolescent patients, the pain can be both cyclic and acyclic [13,15]. In early reproductive ages, the conventionally reported symptoms of endometriosis such as dysmenorrhea and dyschezia are often complemented by less typical vague abdominal, gastrointestinal and genitourinary symptoms [8]. It has been shown that only 9.4% of adolescents experience the classic symptoms of cyclic pain [5,7].

Moreover, the stage of the disease does not clearly correlate with the presence or severity of symptoms, and reciprocally, none of the symptoms are endometriosis-specific [7]. None of the studied candidate biomarkers (endometrium properties, menstrual/uterine fluid and urine composition, immunological parameters in blood, etc.) have shown significant diagnostic value in endometriosis [16]. Given the chronic nature of the disease and the significant impact on reproductive function, ovarian reserve, social and psychological status and the quality of life of young patients, the current main task is to detect the disease as early as possible and to start pathogenetic therapy in a timely manner [10,17].

At the same time, instrumental diagnostics, concerning pelvic ultrasound, is still not accurate enough in the cases of superficial endometriosis [2,18,19]. The advancements in noninvasive diagnosis with respect to imaging modalities have been reported mainly for deep endometriosis with 83–91% sensitivity and 98% specificity for ultrasound and MRI [9]. Superficial noninvasive endometrioses with tiny implants less than 2–3 mm in size on the surface of the peritoneum are often below the resolution of MRI and are not commonly visualized [20]. It is generally recognized that imaging evaluation could be particularly difficult in the early stages of peritoneal endometriosis, and it requires experience and specific training in this field [1,11]. 

Histologic verification after laparoscopic surgery remains the gold standard for confirming the diagnosis, although the results of these surveys do not always correlate [12,13,21]. In cases of negative histological reports, the most common finding in endometriosis of I-II stages is fibrous tissue, with no evidence of glandular epithelium or endometrial stromal cells [11], which determines the difficulty in detecting the initial stages of the disease and timely treatment. Thus, despite the growing number of studies, an appropriate early diagnosis is still a challenge. 

The aim of the current study is to compare the clinical features, instrumental diagnostics and surgical and histological peculiarities in adolescent patients with peritoneal endometriosis. 

## 2. Materials and Methods

The case–control study enrolled girls from menarche to 17 years old (16.0 (15.0–17.0)), with a confirmed diagnosis of peritoneal endometriosis (PE) (main group, *n* = 90) and 24/26.7% of them also having diffuse adenomyosis. All participants underwent inpatient treatments in 2020–2022 in the Pediatric and Adolescent Gynecology Department at the V.I. Kulakov National Medical Research Center for Obstetrics, Gynecology and Perinatology, Moscow, Russia.

The inclusion criteria in the main group were as follows: age of patients from menarche to 17 years inclusive; complaints of persistent moderate–severe dysmenorrhea or/and chronic pelvic pain and resistance to NSAIDs and antispasmodics for at least 3 months preceding the study in the absence of other drug administration (psychotropic and any hormonal drugs, including combined oral contraceptives); laparoscopically confirmed diagnosis of peritoneal endometriosis; informed consent of the patient and her legal representative for participation in the research study. The indications for laparoscopy were as follows: unsuccessful empirical treatment (NSAIDs, antispasmodics and COCs) or persistent moderate/severe dysmenorrhea with negative imaging results, ultrasound or MRI signs of deep infiltrative endometriosis. 

The exclusion criteria were as follows: age over 18; aggravated chronic or acute diseases (infectious, endocrine, oncological, etc.); mental conditions; pelvic tumors; absence of dysmenorrhea and/or chronic pelvic pain (for the main group); hereditary syndromes and congenital malformations associated with menstrual outflow obstruction; lack of informed consent.

The control group consisted of 44 healthy adolescent girls of the same age (16.0 (15.0–17.0)) with regular periods and no gynecological and endocrine pathologies in the absence of routine drug administration. The exclusion criteria were mostly the same with the main group: age over 18; an aggravation of chronic or acute somatic and/or infectious disease; mental illnesses; endocrine or gynecological disorders; tumors of the pelvic organs; oncological diseases; secondary dysmenorrhea and/or chronic pelvic pain; inherited syndromes and congenital malformations associated with an obstruction of menstrual blood outflow; lack of informed consent of the patient or her legal representative for participation in the research study.

General clinical examination was performed for all participants. It included a detailed medical history, heredity peculiarities, anthropometric data (height, weight and BMI) and menstrual cycle characteristics. Pain sensations were assessed using the visual analog pain rating scale (VAS)—a 10 cm horizontal straight segment with each centimeter adding a point from “no pain” (0 points) on the left to “unbearable pain” (10 points) on the right. The patients were asked to make a mark on the scale in order to rate their experience of pain intensity; the outcomes were classified as mild (0–2 points), moderate (2–4 points), severe (4–6 points), very strong (6–8 points) or unbearable (8–10 points).

In the biochemical profile, the concentrations of total protein, uric acid, creatinine, direct and total bilirubin, glucose, Ca^2+^, Fe^2+/3+^ and highly sensitive C-reactive protein (CRP) in venous blood were determined for all participants. 

On the 3rd to 4th day of a spontaneous menstrual cycle, all girls included in the study were subjected to an extended analysis of the blood hormonal profile: The levels of luteinizing hormone (LH), thyroid-stimulating hormone (TSH), thyroxine, follicle-stimulating hormone (FSH), dehydroepiandrosterone sulfate (DHEAS), androstenedione, prolactin (Prl), estradiol (E2), cortisol, testosterone (T), antibodies to thyroid peroxidase (AT-TPO) and sex hormone-binding globulin (SHBG) and tumor markers Ca-125 and Ca-19-9 were determined. Hormonal assays were carried out by electro- and immunochemiluminiscent methods on Cobas e 411 (F. Hoffmann-La Roche, Basel, Switzerland), Immulite 2000 and Immulite 1000 (Siemens, Los Angeles, CA, USA) automatic analyzers using reagents of the same companies. The concentrations of anti-Mullerian hormone (AMH) and 17-OH-progesterone (17-OHP) were measured by the enzyme-linked immunoassay on the DYNEX DSX System analyzer and using the Diagnostic Products Corporation (DPC) system on the Immulite device (DYNEX Technologies, Chantilly, VA, USA).

All girls underwent an ultrasound examination for the pelvic organs and mammary and thyroid glands on days 3–5 of a spontaneous menstrual cycle. The study was performed on a Vivid-q ultrasonic device from GE HEALTHCARE company (GE Healthcare, Chicago, IL, USA) using a linear and convex probe with a frequency of 1.8–6.0 MHz. The study was conducted with a fully filled bladder using the transabdominal approach. The sonographic features, including the length, width and thickness (L, W and T, respectively) of the left and right ovaries, and uterine (U) measurements (L, W and T) were described.

An MRI of the pelvic organs was performed shortly before menstruation (starting from day 25 of the menstrual cycle). Imaging was carried out in GE Signa Excite 1.5T and GE Signa Architect 3.0T MRI systems. T2-weighted images (T2WIs) in sagittal, axial and coronal projections were obtained using a slice thickness of 0.3–0.6 cm and a field of view of 32–42 cm. T1WI and T1FS methods were applied to identify and compare hemorrhagic and fatty components, particularly in the areas of destructive changes. The scanning protocol involved a diffusion-weighted mode to detect ischemic/necrotic sites and hemorrhages and to exclude neoplastic processes. All images were analyzed by the same radiologist in the department of Radiology and Diagnostics at the V.I. Kulakov National Medical Research Center for Obstetrics, Gynecology and Perinatology.

Data from the laparoscopic picture in the main group included surgical diagnosis and the stage of endometriosis according to the revised American Society for Reproductive Medicine (rASRM) criteria [22]; the type, color, size and area of the lesions and the localization of implants were analyzed. Moreover, we evaluated the data from the histological examination of the surgical material (macro- and microscopic description of the tissue involved in the biopsy). The foci of endometriosis were placed in standard cassettes and then transferred to vials and filled with 10% neutral formalin (pH = 7.2) at a ratio of 1:20. Fixation was carried out for 24 h at room temperature. The material was routed according to the standard scheme using isopropyl alcohol with further fillings in paraffin. With help of a rotary microtome, paraffin sections that were 4–5 µm thick were made. After the sections were deparaffinized, hematoxylin and eosin were used for visual staining. Histological examination was carried out with a Leica DM 2500 binocular microscope by adjusting the lighting according to Keller at a magnification of 40×, 100× and 400×. 

Statistical data analysis was performed using the Statistica 12 software from StatSoft Inc. (Tulsa, OK, USA) and IBM-SPSS Statistics for Windows version 26 (IBM Corp., Armonk, NY, USA). Categorical variables were evaluated with the χ^2^ test. A comparison of variables with a normal distribution in two groups was performed by a parametric Student’s *t*-test, and a mean value (M) and a standard deviation (SD) were calculated. In multiple groups, an analysis of variance (ANOVA) was used. The parameters that did not fit the normal distribution were analyzed using a median (Me) and an interquartile range (Q25–75) and the Mann–Whitney U test. The adjusted odds ratio (OR) with a 95% confidence interval (CI) was evaluated using logistic regression methods to analyze the influence of various risk factors. Correlations were estimated using Spearman’s rank correlation coefficient. To evaluate the impact of a single categorical independent factor on a dependent variable, a one-way ANOVA was performed and factorial ANOVA was used to analyze the interactive effects of several categorical factors. The diagnostic accuracy was assessed with logistic regression models and using the receiver operator characteristic (ROC) curve, calculating the area under the curve (AUC).

## 3. Results

The patients of the main group were significantly more likely to have a family history of gynecological diseases in close relatives (endometriosis, abnormal uterine bleedings and moderate–severe dysmenorrhea) compared to healthy controls (32.2% vs. 9.1%, *p* < 0.001 by χ^2^ test) and pregnancy complications in mothers (threatened miscarriage, toxicosis and preeclampsia) (*p* < 0.05, Table 1).

Anthropometric data for the two groups were similar (BMI 20.5 ± 3.7 vs. 20.3 ± 5.8 kg/m^2^, *p* = 0.54). In particular, girls with PE were significantly younger at menarche (11.8 ± 2.5 years vs. 12.5 ± 1.2 years for the control group, *p* < 0.001) and often had irregular periods and/or heavy menstrual flow (Table 1).

In addition, 25.6% (23/90) of patients in the main group reported blood spotting that occurred mostly mid-cycle (52.2%, 12/23) and/or shortly before menses (the responders could choose more than one answer to this question). In 60.9% (14/23) of the cases, the spotting was brown; in the rest, it was scarlet (21.7%, 5/23) or dark red (17.4%, 4/23). Most patients with intermenstrual spotting (65.2%, 15/23) had PE combined to diffuse adenomyosis.

The main complaints in adolescents with PE were severe dysmenorrhea and/or chronic pelvic pain resistant to NSAIDs and antispasmodics (95.6%, 86/90). Although none of the control group’s participants presented severe dysmenorrhea (*p* < 0.001, χ^2^-test), 15.9% (7/44) reported functional dysmenorrhea with occasional single cases of pain reaching 5–8 points on day 1, and these were effectively treated without recurrence by a single dose of NSAID, which can occur during the physiological menstrual cycle. Patients with PE reported pain of high intensity (7.5 ± 2.1 vs. 2.5 ± 2.3 VAS points, *p* < 0.001) that most often began 1 day before menses and lasted 3 days into menses (58.9%, 53/90). Smaller proportions of the patients reported mid-cycle pain (11.1%, 10/90), pain beginning 3–5 days before menses (8.9%, 8/90), daily pain (15.6%, 14/90) and menstrual pain spanning 3–5 days before and after menses (5.6%, 5/90) (Table 1).

In 45.6% (41/90) of the patients, the pain was localized in the “lower abdomen” area, whereas 22.2% (20/90) reported pain in the “lumbar” area and 13.3% (12/90) complained of diffuse pain without clear localization. The patients also reported groin pain on the left (10.0%; 9/90), groin pain on the right (8.9%; 8/90) and pain in the perineum (4.4%; 4/90) or in the sacrum (1.1%; 1/90) (the responders were asked to recollect sensations experienced up to 3 months prior to the survey and could choose more than one answer).

Most patients (63.3%, 57/90) experienced menstrual pain from menarche, and the rest attributed the time of pain onset to some period after menarche (Table 1). Three patients associated the occurrence of pain at some period after the inflammation of genital organs, and four patients associated the occurrence of pain with abdominal or pelvic surgery (Table 1). In 53.3% (48/90) of the patients, pain sensations were of stable intensity, whereas in 46.7% (42/90), they were aggravated.

The descriptions of painful sensations in the main group varied: 44.4% (40/90) of the patients described the pain as “aching, pulling”, 25.6% (23/90) described the pain as “cramping”, 20.0% (18/90) described the pain as “cutting, stabbing”, 10.0% (9/90) described pain as “pressing” and 1.1% (1/90) described pain as “baking (burning)” (the responders could choose more than one definition). In addition, 38.9% (35/90) of the patients had gastrointestinal symptoms (e.g., nausea, diarrhea and pain during defecation) and 24.4% (22/90) reported urination disorders (dysuria and hematuria) associated with menstruation. The majority of patients (75.6%, 68/90) had reduced everyday activity on menstrual days because of pain, which is defined as weakness, decreased performance and physical activity.

**Figure 1:**
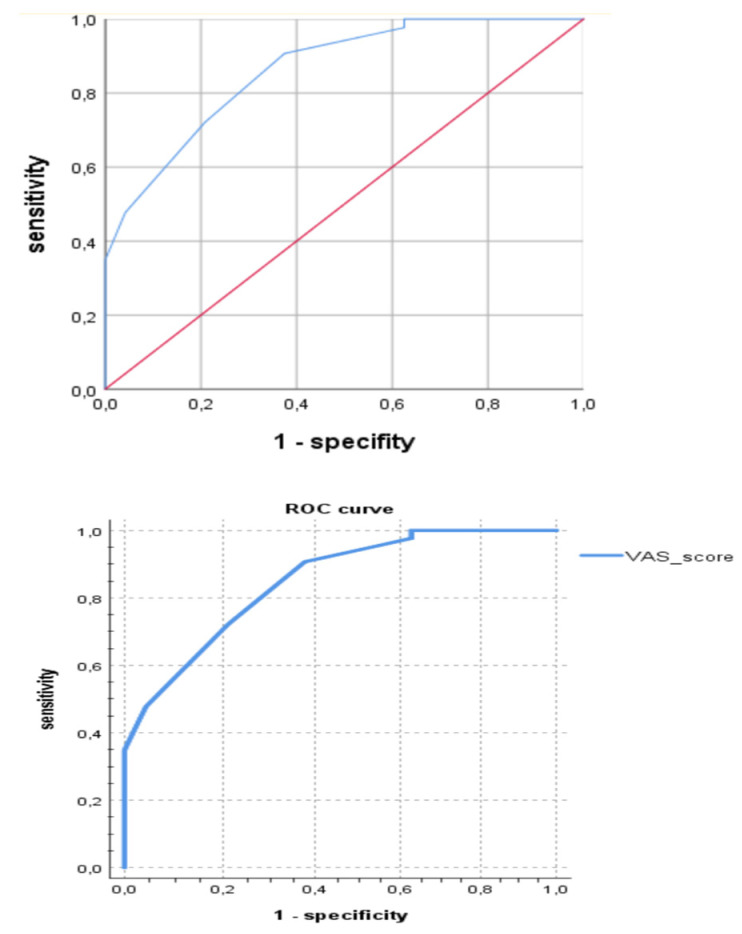
Diagnostic accuracy of VAS score (ROC curve: area under the curve (AUC) = 86.2%, *p <* 0.05) in PE predicting on laparoscopy (baseline is in red, the VAS score line is in blue).

Multivariate analysis confirmed the significance of the family history of endometriosis and other gynecological diseases (abnormal uterine bleeding and dysmenorrhea) in close relatives (OR 4.85, CI = 1.58; 14.87, *p* = 0.005) and dysmenorrhea from menarche (OR 19.30, CI = 7.19; 51.60, *p* < 0.001) as risk factors for juvenile PE. Among clinical and anamnestic factors, the highest diagnostic accuracy for PE in adolescents was shown for persistent dysmenorrhea (80.9%, χ^2^ = 19.54, *p* < 0.001) and VAS score (91.8%, χ^2^ = 65.11, *p* < 0.001). The ROC analysis revealed the sensitivity of 71.8% and a specifity of 79.2% for the cut-off VAS score in 6.50 points in the prediction of PE on laparoscopy (AUC = 0.862, *p* < 0.05) (see Figure 1). 

Routine blood tests revealed no between-the-group differences, apart from slightly higher eosinophil counts in patients with PE compared to healthy controls (10^8^/L; 2.6 ± 2.2 vs. 1.9 ± 2.1, *p* = 0.04). The measured values of other inflammation markers including total WBC counts (10^9^/L; 6.5 ± 1.8 vs. 6.9 ± 2.4, *p* = 0.4), sedimentation rate of erythrocytes (ESR) (cm/h; 3.4 ± 2.2 vs. 3.3 ± 1.6, *p* = 0.8) and C-reactive protein (CRP, mg/L; 0.9 ± 0.9 versus 1.0 ± 0.6, *p* = 0.9) were similar.

Hormone profiling revealed higher levels of LH, estradiol, 17-OH progesterone and prolactin in patients with PE compared to the control group, albeit within reference laboratory values (Table 2). Multivariate analyses identified higher levels of LH (OR 0.09, CI = 0.02; 0.37, *p* < 0.001) as a risk factor for PE in adolescents.

The levels of CA-125 in patients with PE were higher than in the control group (U/mL; 31.8 ± 56.1 vs. 19.0 ± 10.2, *p* = 0.006), albeit not exceeding the upper reference limit; the differences for other tumor markers were non-significant.

In sonographic images, patients with PE presented with thicker uterine walls (cm; 3.3 ± 0.8 vs. 2.9 ± 0.5, *p* = 0.001) and thicker endometrium (cm; 0.8 ± 0.3 vs. 0.5 ± 0.3, *p* < 0.001) compared to the control group, whereas ovarian morphologies were similar. The signs of PE and adenomyosis were encountered in only 3.3% (3/90) and 20.8% (5/24) of the cases with corresponding final diagnoses.

**Figure 2:**
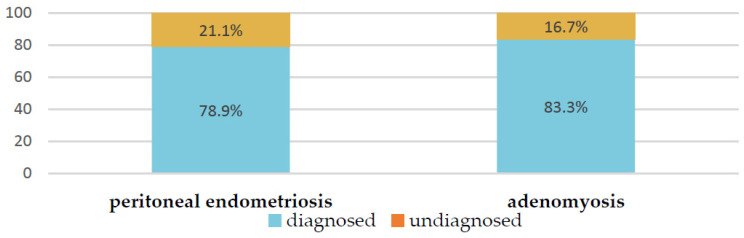
Frequency of suspected PE and adenomyosis according to MRI in adolescents.

By contrast, the MRI resulted in a suspected diagnosis of adenomyosis in 83.3% (20/24) and PE in 78.9% (71/90) of cases with corresponding final diagnoses (Figure 2). The other 21.1% (19/90) of the PE cases, suspected on the basis of severe persistent dysmenorrhea but unsupported by MRI, were eventually confirmed by laparoscopy.

**Figure 3:**
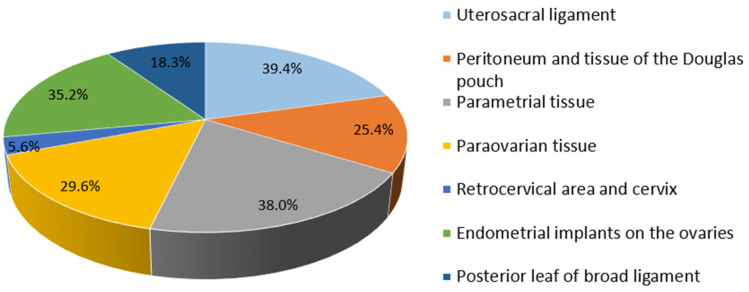
Localization of endometriotic foci according to MRI in adolescents.

**Figure 4:**
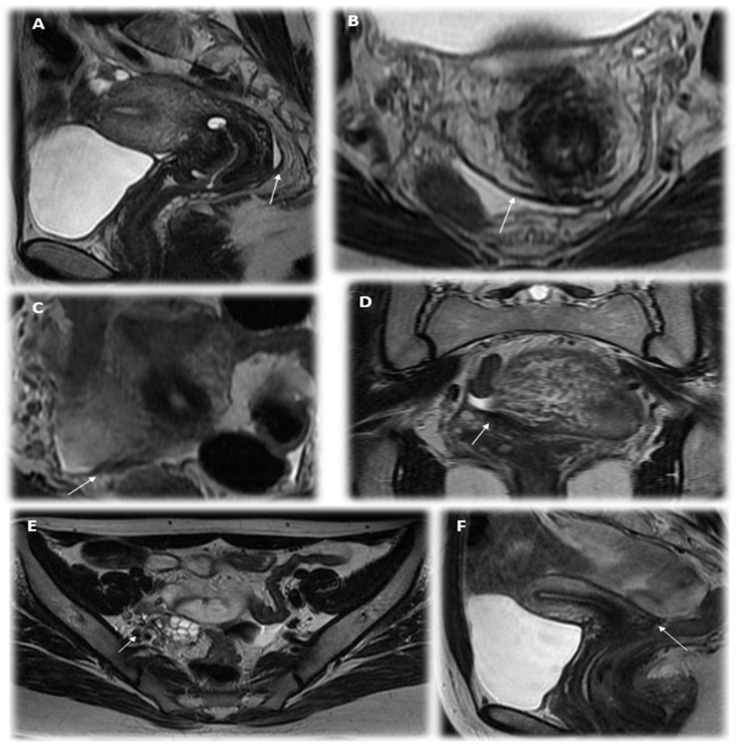
The MRI signs of peritoneal endometriosis in adolescents: (**A**)—thickening of the peritoneum and partial obliteration of the pouch of Douglas (T2VI Sag); (**B**)—thickening of the peritoneum of the Douglas space, free fluid and an area of low intensity of the MR signal in the projection of the uterosacral ligament on the left (T2VI Ax); (**C**)—the thickening of the pelvic peritoneum and the uterosacral ligament on the right (T2VI Ax); (**D**)—thickening of the peritoneum of the pelvic cavity and the right uterosacral ligament (T2WI Cor); (**E**)—the asymmetry of the structure and hypointense MR signal in the projection of the sacro-uterine ligament on the right and fixation of the right ovary (T2VI Ax); (**F**)—a single focus of a hypointense MR signal in the retrocervical space of small sizes (up to 0.3 cm) (T2VI Cor).

We paid special attention to the analysis of the MRI’s description in the cases of PE (Figure 2). Of the 78.9% (71) patients with verified PE according to MRI, in most cases, lesions were described on the peritoneum of the pouch of Douglas (25.4%); along the serous cover of the uterus (posterior leaf of the broad ligaments) (18.3%); on the uterosacral ligaments (39.4%); in the parametrial (38.0%), paraovarian (29.6%) and paracervical (5.6%) tissue; and suspected along the ovarian capsule (35.2%). As additional parameters, the meager amount of free fluid in the retrouterine space (63.3%; 45/71); adhesions in the pelvis, fixation of the fallopian tube, intestine and/or ovaries (32.4%; 23/71); and varicose veins of the pelvis (33.8%; 24/71) were described. These indicators could also be useful in diagnostics (see Figure 3, Table 3 and Table 4). We illustrated the most essential MRI signs in Figure 4.

We analyzed the significance of detected MRI signs in predicting the confirmation of PE according to laparoscopy in adolescents using factor analyses. The most essential MRI signs of PE appeared to be the following: heterogeneity of paraovarian, parametrial or paracervical tissue or/and hypointense foci in the pelvic tissue, thickening or flattening of the uterosacral ligaments or asymmetric nodular irregularity of ligaments, thickening of the peritoneum and/or heterogeneity of the tissue of the pouch of Douglas, the presence of a meager amount of free fluid in the rectouterine pouch, adhesions in the pelvis and fixation of the fallopian tube, intestine and/or ovaries (see Table 3).

**Figure 5:**
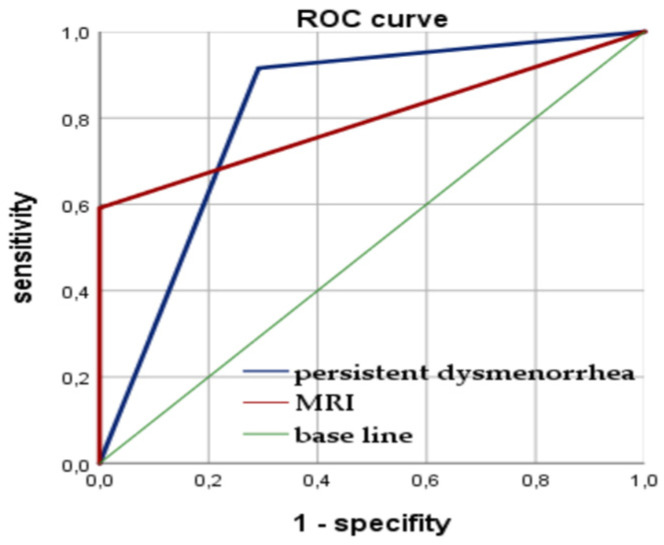
Diagnostic accuracy of MRI signs (ROC curve: area under the curve (AUC) = 0.796, *p <* 0.05) and persistent dysmenorrhea (AUC) = 0.812, *p <* 0.05) in predicting PE.

One of two MRI signs for ligaments or/and for the heterogeneity of the pelvic tissue provides a diagnostic accuracy of PE in 74.7% (F = 7.2, *p* = 0.008; area under the curve (AUC) = 0.796, *p <* 0.05), which could be helpful in early diagnostics (Figure 4), although it would not be enough for practical application while making a decision on whether to perform diagnostic laparoscopy. Thus, we analyzed the probability of detecting PE using laparoscopy not only according to the MRI criteria but also in combination with the clinical symptoms of persistent dysmenorrhea. The diagnostic accuracy of the parameter of persistent dysmenorrhea in predicting PE was also rather high. It appeared due to the combined use of two criteria: the complaint of persistent dysmenorrhea and positive MRI signs results in a higher accuracy of predicting the initial from of PE at 84.3% according to multivariate analysis (logistic regression model: OR 15.4, Wald’s Chi-square = 32.7, <0.001; confirmed with factorial ANOVA: F = 10.2, *p* = 0.002) than with the use of each index separately; the ROC analysis is presented in Figure 5. The diagnostic model, based on these parameters (persisted dysmenorrhea and one of two MRI signs), can be suggested for applications, justifying the early surgical diagnostics, shortening the time delay and suffering of the young patients and resulting in required therapy.

The laparoscopic picture of patients with peritoneal endometriosis was analyzed and hereinafter compared with MRI data. The I stage of endometriosis according to the revised American Society for Reproductive Medicine (rASRM) score was detected in the majority of adolescents (64.4%, 58/90, the rASRM score was 3.1 ± 1.3); the II stage—in almost one-third of patients (27.8%, 25/90, the rASRM score was 8.4 ± 3.1); and the III stage was observed in 7.8% cases due to endometriomas (7/90, the rASRM score was 24.5 ± 7.5).

According to laparoscopy, a different localization of PE foci was noted (see Table 3). Endometrial lesions were described predominantly in the uterosacral ligaments (84.4%, 76/90) and the pouch of Douglas (66.7%, 60/90); in more than in one-third of girls, foci were detected in the peritoneum of the ovarian fossae (38.9%, 35/90) and approximately equally on the plica vesicauterina (26.7%, 24/90) and retrocervical region (25.6%, 23/90); thus, the different compartments of the pelvis were already involved in the disease during adolescence.

A comparison of the data of MRI and laparoscopy in relation to PE localization in adolescents revealed that the MRI suggested the direct compartment of lesions in less than half of the cases (in 35.6%, 28/76) on the uterosacral ligaments and in one-third of cases on the peritoneum of the pouch of Douglas (in 30.0% (18/60). The visualization results almost matched for the lesions in the paraovarian tissue and the peritoneum of ovarian fossa (60.0%, 21/35). However, the MRI showed false-positive results when the lesions were located in the retrocervical area (27 cases from 24 due to LS) and the serosa of the uterus (13 from 2 cases) (see Table 4).

**Figure 6:**
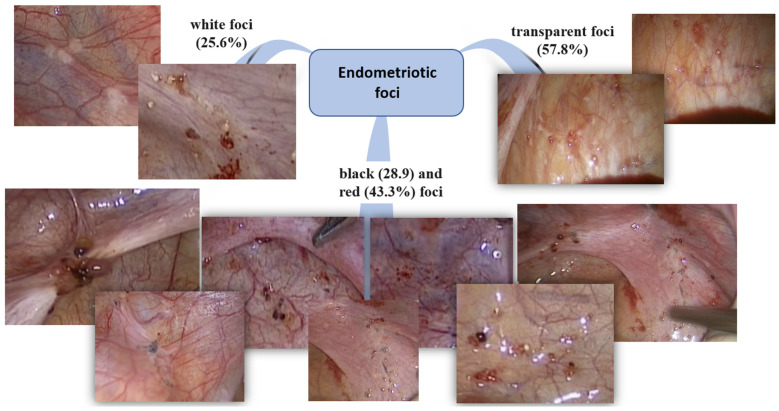
Characteristics and the color of endometriotic foci in adolescent patients in the study’s sample.

In most cases, patients had transparent confluent foci (57.8%, 52/90); in a third of patients, red (43.3%, 39/90) and black foci (28.9, 26/90%) and white fibrous foci (25.6%, 23/90) were observed. In 14.4% (13/90) of patients with prominent vasculature, adhesions (15.6%, 14/90) and serous hemorrhagic fluid in the pouch of Douglas (63.3%, 57/90) were observed. Schematically, the features of endometriotic foci are presented in Figure 6. Red lesions were more likely observed at a more severe rASRM stage (r = 0.27, *p* = 0.017. Transparent lesions were associated with a higher VAS score (r = 0.32, *p* = 0.007). White foci correlated with prominent vasculature (r = 0.24, *p* = 0.033).

Furthermore, the histological findings were evaluated. In 67.8% (61/90), lesions were represented by endometrial-like tissues (44.4% endometrial type glands, and in 25.6%, endometrial stroma was detected); in addition, the areas of fibrous (100%), adipose (44.4%), muscle (31.1%) tissue, hemorrhages (7.8%), vessels (7.7%), the areas of calcification (3.3%) and infiltration with polynuclear leukocytes (5.6%) were described. In one of the cases, a combination of endosalpingiosis and endometrial-like tissues was determined (2.2%). However, in 32.2% (29/90) of cases, endometrial-like tissues were not identified. In these cases, the foci of endometriosis were represented mainly by fibrous (73.3%), adipose (31.1%), muscle (21.1%) tissue and areas of hemorrhage (21.1%); in 20.0% (18/90) of cases, the tubal epithelium was visualized, which determined the histological conclusion of endosalpingiosis.

Black lesions were more likely to have histological verification (r = 0.41, *p* = 0.001). In addition, plethoric and developed vessels in the histological picture were associated with a higher rate of detection of polynuclear leukocytes (r = 0.48 and r = 0.39, *p* = 001, respectively). 

Thus, in the third part of cases, no endometrial glands or stromal cells were detected; however, such a histological picture does not correspond to a healthy peritoneum and does not exclude the diagnosis according to the ESHRE 2022 recommendations [23].

## 4. Discussion

Endometriosis is the most common reason for persistent dysmenorrhea and chronic pelvic pain in adolescents. At the same time, there are very few clinical studies on endometriosis in adolescents in the available literature; thus, the management strategy and tactics are customarily extrapolated from data obtained in adults [17,24]. It is known that hereditary predisposition, early age of menarche (<14 years), short menstrual cycle, longer menstrual bleeding, obesity and the early onset of dysmenorrhea are the risk factors for endometriosis [23,25,26]. Here, we show that girls with a first-degree relative with endometriosis or related gynecological conditions (abnormal uterine bleeding and dysmenorrhea) have a 4.9-fold increased risk of endometriosis (CI 1.58–14.87, *p* = 0.005), consistent with other studies, and evidenced a polygenic multifactorial inheritance pattern relative to endometriosis with a heritability of 51–71% [2]. 

A meta-analysis of 18 publications investigating the association between menarcheal age and endometriosis revealed a weak positive correlation between endometriosis and early menarche [27], possibly reflecting the early activation of the hypothalamic–pituitary–ovarian axis, an increase in the overall number of periodic bleedings and the concomitantly elevated risks of the retrograde reflux of menstrual discharge to the abdominal cavity [7]. In our settings, girls with PE were on average 8 months younger at menarche than healthy controls (11.8 ± 2.49 vs. 12.5 ± 1.19 years, *p* < 0.001 by *t*-test).

The relationship between menstrual cycle length and endometriosis is complex [2]. Both longer and shorter menstrual cycles (compared to the 28 days reference) have been associated with higher risks of the disease [1,8]. Although here we reveal no between-the-group difference in menstrual cycle length, girls with PE were significantly more likely to report irregular and heavy bleedings amid a hyperestrogenic status, consistently with the conjecture of a higher risk of menstrual reflux during a prolonged and heavy period [2].

Concerning pelvic pain, it is reported that adolescents express severe dysmenorrhea and acyclic nonclassical pain mostly for a prolonged time from menarche [15,28,29]. Our results confirm that such pains (occasionally reaching ‘unbearable’ values) are characteristic for the condition. In about one-third of the cases, pain is experienced almost daily and often accompanied by gastrointestinal and urinary symptoms, which strongly affects emotional and physical well-being, everyday activity and individual performance. Very obviously, such cases require early diagnosis and management in order to preserve both the health and the quality of life in a young girl starting from the age of initial presentation with the disease [30]. It is underlined in previous studies that the pattern of pelvic pain in adolescents with endometriosis differs from the pattern in adults [9,31]. If adults usually experience pain, it precedes the onset of the menses with an increase during menstruation; adolescents indicate acyclic pain and other pain-related symptoms that can occur unpredictably or continuously throughout the menstrual cycle, and this could be worthy to consider as a reliable clinical factor for diagnoses.

An important developmental facet in endometriosis pathogenesis is linked to the maturation of hypothalamic–pituitary projections during puberty. The higher systemic levels of luteinizing hormone (LH) and prolactin observed in the main group of the study may serve as a predisposing factor in girls with the delayed maturation of the hypothalamic-–pituitary system and elevated volume/frequency of LH release. In addition, endometriosis is a hormone-mediated condition [2], and according to our results, the patients presented higher levels of estradiol, 17-OH progesterone and total androstenedione, which also implicates the hypothalamic–pituitary connection. In addition, a higher level of estradiol may predispose the individual to the disease’s progression and chronic inflammatory reactions in the endometrial tissue in an estrogen-dependent pathway.

Adolescent endometriosis is generally underestimated; as the imaging evaluation remains challenging, transvaginal ultrasound and vaginal examination could be contraindicated [9,31]. There is debate on the requirement for pre-operative ultrasounds [2]. Authors underline that ultrasound data could be normal in adolescents presenting the initial stages of endometriosis, as superficial endometriosis may not be visualized, and that does not rule out the disease [2,7]. Despite the continual improvement of imaging techniques and digital tools for data interpretation, pelvic sonography provides low diagnostic accuracy for endometriosis—except for advanced stages (ovarian endometriomas and deep infiltrative lesions) that are rarely found in adolescents [8,14,18]. In our setting, the ultrasound examination provided unsatisfactory diagnostic means for peritoneal endometriosis. Moreover, the method showed <50% sensitivity for adenomyosis, probably due to a less conspicuous nature of this pathology in adolescents compared to older patients.

MRI, by contrast, provides rather accurate diagnostic means for endometriosis including deep infiltrative forms, and these are informative in terms of organ involvement and adhesive process severity [32]. Superficial peritoneal implants < 5 mm in diameter are formally below the resolution of MRI; still, a tentative search for the presence of small T1-weighted hyperintense foci may provide crucial grounds for the diagnosis [20]. Our data confirm the fundamentally higher diagnostic accuracy for PE achieved with MRI compared to ultrasonography. The MRI signs of PE evaluated by us here (including heterogeneity and hypointense foci in paraovarian/parametrial/paracervical tissue, thickening/flattening of uterosacral ligaments and irregular homogeneity of rectouterine lining) are eligible for the early diagnosis of PE in adolescents.

Laparoscopic evidence provides a gold-standard verification for PE, determining the stage, spread and severity of the process [33]. Endometrial lesions in adolescents often look different than those in adults [34]. Generally, for the given age group, confluent vesicular or papular, clear, red and white lesions are more typical than black, blue or powder burn lesions [15]. In our study, laparoscopy most often revealed multiple multicolored foci: clear (57.8%), red (43.3%), black/cyanotic (28.9%) and white (25.6% of the cases). Clear, white and red foci positively correlated with, respectively, the VAS score of pain intensity, the degree of vascularization and rASRM score. Such foci are classified as ‘dynamic’ lesions with active prostaglandin synthesis contributing to noxious stimuli [35]. Red or clear nonpigmented lesions are considered to be more biologically active than black lesions; such implants are associated with pronounced vascularization in the absence of local sclerosis, and they are more commonly found in adolescent than adult patients [7].

The majority of our patients presented with stage I or II of the disease (64.4% and 27.8% and rASRM scores of 3.1 ± 1.3 and 8.4 ± 3.1, respectively), and a minority presented stage III (7.8%, 24.5 ± 7.5). The lesions were localized predominantly in uterosacral ligaments (84.4% of the cases) and the pouch of Douglas (66.7% of the cases). These data are consistent with other studies: Smorgick et al. reported early stages I/II in 76% of the patients, with the rest having advanced stages III/IV% [6]. A study by Matalliotakis et al. enrolled 55 adolescents with endometriosis stage I, II, III and IV in, respectively, 45.4%, 36.4%, 14.5% and 3.7% of the cases [5]. Similarly, the systematic review by Janssen al. classified the disease as minimal, mild, moderate or severe in, respectively, 50%, 27%, 18% and 14% of adolescents [3].

Back in 1987, a study by Chatman and Zbella enrolling 115 patients with endometriosis observed that conspicuous, clinically and laparoscopically substantiated diagnoses (characteristic symptoms and macroscopic lesions) often fail to be supported histologically [36]. In modern practice, the diagnosis of endometriosis chiefly relies on clinical data and only secondarily on surgical and histological findings [37]. In a prospective study by Stratton et al. enrolling 65 patients with a total of 314 foci identified and excised, only 61% of the cases were histologically ‘positive’; moreover, the presence of endometrial-like patterns was attributed with lower diagnostic significance than gross morphology (diameter and depth) of the lesions [38]. At that, the probability of histological confirmation was only vaguely related to the localization/color of the foci, whereas superficial and small lesions were less likely to be histologically ‘positive’ than larger affected areas. Marchino et al. assessed 122 biopsies collected from 54 patients with endometriosis, histologically confirming the disease in 54% of the lesions—via the observation of glandular and stromal components of endometrial-like tissue [39]. For classical lesions, the histological compliance was higher than for atypical lesions (mostly fibrotic). In our setting, the discrepancy between the laparoscopic visualization and histological identification of endometrial-like components was high: In 32.2% of the cases, no endometrial-like tissues were found in the biopsies; instead, the foci contained various proportions of fibrous, adipose and muscle tissues with or without signs of hemorrhage. Hence, histological verification for the initial stages of peritoneal endometriosis remains often challenging, as the endometrial-like structures in peritoneal lesions can be poorly distinguishable or disappear. This obstacle also could be overcome with more information provided to the laboratory and the experience of a pathologist who is accustomed to looking for disease lesions [38].

According to published evidence, the endometrial foci of white/mixed color are more likely to contain endometrial-like morphological patterns; accordingly, black or red lesions are less often confirmed histologically [38]. In this study, we found no association between the external color and the presence of endometrial-like tissue in the foci. At the same time, black foci positively correlated with severity (r = 0.382, *p* = 0.045) and AFS score (r = 0.443, *p* = 0.018). El Bishry G. et al. previously reported a correlation between histological compliance and severity [11]; however, it was not reproduced in our setting (r = −0.096, *p* = 0.626) probably due to the specific structure of the cohort (adolescent patients manifesting the predominantly initial forms of the disease). The lack of association between clinical stage and dysmenorrhea severity as assessed by VAS (r = 0.205, *p* = 0.483) is consistent with other studies [1,5]. 

It should be emphasized that the decision on pelvic laparoscopy in an adolescent is a fundamentally sensitive issue: on the one hand, such decisions should imply that all conservative options have been exhausted; on the other hand, postponing may lead to uncontrollable disease progression with disastrous consequences [10,35].

Considering the substantial impact of endometriosis on social and psychological well-being, as well as the progressive nature of the disease and its damaging effect on reproduction, early diagnosis and management are pivotal. Despite the lack of reliable biomarkers and noninvasive diagnostic criteria, a comprehension of family history, clinical status and MRI data by an experienced specialist can result in the recognition of juvenile endometriosis with high accuracy [40]. Laparoscopic surgery is indicated in young patients with chronic pelvic pains and dysmenorrhea resistant to conservative treatments. The absence of specific signs in pre-operative MRI and ‘negative’ histology cannot exclude peritoneal endometriosis in an adolescent, which justifies early surgical management in certain cases of persistent heavy dysmenorrhea in young patients.

## 5. Conclusions

The main conclusions from the study are formulated as follows:

1. A family history of endometriosis and dysmenorrhea from menarche increases the risks of peritoneal endometriosis (PE) in a girl, respectively, 5- and 20-fold with 95% CIs of 1.58–14.87 and 7.19–51.60 (*p* ≤ 0.005).
2. The main endometriosis-associated complaints in adolescents are moderate–severe dysmenorrhea (VAS score 7.5 ± 2.1), decreased everyday activity/performance (75.6%), gastrointestinal (38.9%) or urinary (22.4%) symptoms and mid-cycle spotting (25.6%). Persistent dysmenorrhea (80.9%, χ^2^ = 19.54, *p* < 0.001) and VAS score (91.8%, χ^2^ = 65.11, *p* < 0.001) show the high diagnostic significance for PE in adolescents.
3. Patients with PE have higher plasma levels of luteinizing hormone, prolactin, estradiol, 17-OH progesterone and CA-125 (<0.05 for each). Ultrasonography has a low diagnostic potential for both PE and adenomyosis in adolescents.
4. Significant MRI signs for the peritoneal form of the disease include the heterogeneity of paraovarian or parametrial tissue and/or hypointense foci in the tissue and the pouch of Douglas and the thickening or nodular irregularity of uterosacral ligaments or uterine serosa (<0.05 for each). The positive MRI signs for ligaments and/or the pelvic tissue heterogeneity provided 74.7% (*p* = 0.008) diagnostic accuracy on their own. However, the fundamental limitations of MRI as (so far) the best diagnostic approach for the initial stages of peritoneal endometriosis motivate laparoscopic interventions upon the indication of persistent pelvic pain and severe dysmenorrhea in adolescents, at least in MRI-negative cases.
5. The majority of adolescent patients with PE (64.4%) had stage I (rASRM score 3.1 ± 1.3), followed prevalently by stage II (27.8%, 8.4 ± 3.1) and stage III (7.8%, 24.5 ± 7.5). Peritoneal lesions were found predominantly in uterosacral ligaments and the pouch of Douglas (84.4% and 66.7%, respectively). Clear vesical, red and white foci (observed in, respectively, 57.8%, 43.3% and 25.6% of the cases) correlated with the VAS score for pain intensity (r = 0.32, *p* = 0.007), rASRM score for severity (r = 0.27, *p* = 0.017) and degree of vascularization (r = 0.24, *p* = 0.033), respectively.
6. The combination of persistent dysmenorrhea and MRI signs for ligaments and/or pelvic tissue heterogeneity provided high diagnostic accuracy for PE detection in adolescents (80.9%, χ^2^ = 19.54, *p* < 0.001).

## Figures and Tables

**Table 1:** Anamnesis, menstrual cycle and clinical characteristics in adolescents with endometriosis and “healthy” girls.

Parameters	Endometriosis (*n* = 90)	Control Group (*n* = 44)	*p*-Value
Family history (endometriosis, moderate–severe dysmenorrhea, uterine bleeding) *	32.20 (29)	9.09 (4)	<0.001
Pregnancy complications in mother *	77.78 (70)	36.36 (16)	0.047
Age of menarche ^^^ (years)	11.8 ± 2.5	12.5 ± 1.2	<0.001
Spotting # In the middle of the cycle 2–3 days before menses 3–5 days before and after each menstruation	25.6% (23) 52.2% (12) 34.8% (8) 17.4% (4)	0	<0.001 <0.001 <0.001 0.003
Amount of menstrual blood loss # Light Moderate Heavy	3.33 (3) 62.22 (56) 34.44 (31)	9.09 (4) 90.90 (40) 2.27 (1)	0.159 0.001 <0.001
Duration of menstruation (days) * <5 5–7 >7	26.67 (24) 62.22 (56) 11.11 (10)	40.90 (18) 54.54 (24) 4.45 (2)	0.096 0.394 0.205
Onset of dysmenorrhea # At menarche Within 6 months post-menarche A year—1.5-year post-menarche After pelvic inflammatory disease After abdominal/pelvic surgery	63.3% (57) 7.8% (6) 22.2% (20) 3.3% (3) 4.4% (4)	9.9% (4) 2.2% (1) 15.9% (7) 0 0	<0.001 0.199 0.393 0.223 0.158
Pelvic pain experienced #: On days −1 … till 3 of the cycle Almost every day/not related to cycle In the middle of the cycle 3–5 days before the onset of menses and lasts several days 3–5 days before menses, throughout menses and 3–5 days more	58.9% (53) 15.6% (14) 11.1% (10) 8.9% (8) 5.6% (5)	11.4% (5) 0 4.5% (2) 0 0	<0.001 0.548 0.776 0.214 0.733
Persistent to NSAIDs dysmenorrhea #	95.6% (86)	9.1% (4)	<0.001
Moderate–severe dysmenorrhea #	97.8% (88)	15.9% (7)	<0.001
Severity of menstrual pain (visual analogue score) ^^^	7.5 ± 2.1	2.5 ± 2.3	<0.001
Decreased daily activity and performance #	75.6% (68)	9.1% (4)	<0.001
Gastrointestinal pain symptoms # (nausea, diarrhea, painful bowel movements)	38.9% (35)	6.8% (3)	<0.001
Urinary symptoms * (dysuria, hematuria)	22.4% (22)	0	<0.001
Regular menstrual cycle * Established from the 1st cycle Established within the year after menarche Not established	62.22 (56) 13.33 (12) 24.44 (22)	75.0 (33) 20.45 (9) 4.45 (2)	0.216 0.663 0.519
Regularity * (within 24–38 days) Regular Irregular	55.56 (50) 44.44 (40)	84.09 (37) 15.91 (7)	0.049 0.155
The duration of the menstrual cycle (days) * 21–28 29–35 36–45	52.22 (47) 45.56 (41) 2.22 (2)	31.81 (14) 56.81 (25) 11.36 (5)	0.180 0.375 0.699

* Calculation was made using Pearson’s χ^2^ criterion; # calculated using the χ^2^ test with Yates correction; ^ Student’s *t*-test.

**Table 2:** Plasma levels of hormones and tumor markers in adolescents with endometriosis and “healthy” girls.

Parameters	Endometriosis (*n* = 90)	Control Group (*n* = 44)	*p*-Value
LH **, IU/L	6.70 (4.60–9.10)	3.68 (2.51–4.73)	<0.001
FSH *, IU/L	5.30 (4.10–6.55)	5.20 (4.02–6.80)	0.344
TSH **, mIU/L	2.09 (1.30–2.66)	1.97 (1.54–2.29)	0.561
T4 free **, pmol/L	14.70 (13.00–15.40)	13.80 (13.30–13.90)	0.579
Prolactin **, mIU/L	385.50 (257.00–693.00)	202.00 (149.50–267.00)	0.036
Estradiol **, pmol/L	217.50 (132.00–463.00)	178.00 (116.00–210.00)	0.032
Testosterone **, nmol/L	0.80 (0.53–1.12)	0.94 (0.70–1.14)	0.611
Cortisol **, nmol/L	360.00 (289.25–427.00)	404.17 ± 100.59	0.539
AMH **, ng/mL	4.35 (3.68–8.30)	5.70 (3.30–6.80)	0.162
Androstenedione **, ng/mL	10.60 (8.74–12.65)	8.11 (6.45–10.60)	0.053
AT-TPO **, ME/мл	10.80 (8.00–17.70)	14.30 (8.00–20.60)	0.933
17-OHP **, nmol/L	5.20 (3.90–8.00)	3.45 (2.80–4.10)	0.022
DHEAS **, μmol/L	5.54 (3.77–7.06)	5.42 (4.14–5.72)	0.719
SHBG *, nmol/L	60.85 ± 24.37	64.70 ± 33.48	0.257
CA-125 **, U/mL	20.0 (14.06–32.15)	13.82 (11.45–17.67)	0.049
CA-19.9 *, U/mL	20.59 ± 29.94	15.07 ± 20.67	0.688
antigen HE4 *, pmol/L	49.82 ± 9.51	50.42 ± 11.72	0.848
CEA *, ng/mL	0.89 ± 0.49	1.82 ± 0.94	0.096
α-Fetoprotein *, IU/mL	1.08 ± 0.31	1.40 ± 0.26	0.194
hCG *, IU/L	84.47 ± 40.96	101.0 ± 35.72	0.521
CRP *, mg/L	0.85 ± 0.98	0.99 ± 0.61	0.891

* Normally distributed data presented as mean ± standard deviation, Student’s *t*-test. ** Non-normally distributed data presented as median (25–75 percentiles), Mann–Whitney U test. LH: Luteinizing hormone; TSH: thyroid-stimulating hormone; FSH: follicle-stimulating hormone; SHGB: sex hormone-binding globulin; 17-OHP: 17α-OH progesterone; DHEAS: dehydroepiandrosterone sulfate; AMH: anti-Mullerian hormone; TPO-Ab: antibodies to thyroid peroxidase; CRP: highly sensitive C-reactive protein; CEA: cancer embryonic antigen; HCG: human chorionic gonadotropin.

**Table 3:** Significance of the MRI signs in the prediction of laparoscopic conformation of peritoneal endometriosis in adolescents.

MRI Signs	F	*p*-Level	AUC (%)
Suspicion of peritoneal endometriosis according to MRI (overall)	54.10	<0.001	0.853
Indirect signs of sacro-uterine ligaments endometriosis (nodularity, heterogeneity, nodular irregularity)	11.01	0.002	0.667
Thickening or flattering of sacro-uterine ligaments	6.65	0.012	0.615
Hypointense foci, heterogeneity or signs of endometriosis of pelvic tissue (overall, including rectouterine tissue)	7.45	0.008	0.625
Indirect signs of endometriosis of the peritoneum (thickening, hypointense foci, heterogeneity)	10.37	0.002	0.660
Heterogeneity of paraovarian tissue	8.97	0.004	0.643
Heterogeneity of parametric tissue	8.94	0.004	0.657
Heterogeneity of paracervical tissue	2.19	0.142	0.545
Thickening of the tissue of Douglas space	2.7	0.105	0.554
Fixation of intestine	1.72	0.193	0.536
Fixation of the ovaries (to the pelvic wall)	0.83	0.365	0.518
Thickening of the fallopian tubes	3.2	0.077	0.563
Thickening of the capsule of the ovary	12.45	<0.001	0.679
Signs of endometriosis of the serous cover of the uterus	4.91	0.029	0.509
Salpingo-ophoritis	4.82	0.031	0.606
Meager amount of free fluid in small pelvis	19.85	<0.001	0.764
Adhesions in the pelvis	10.53	0.002	0.660
Varicose veins in the small pelvis	8.62	0.004	0.639

Univariate tests of significance (F) and ROC analysis results (area under the curve, AUC).

**Table 4:** Localization of endometriotic foci according to MRI and laparoscopy (LS) in the study’s sample.

Foci Localization	According to MRI %. (*n* = 71)	According to LS %. (*n* = 90)	*p*-Level
Uterosacral ligament *	39.4 (28)	84.4 (76)	*p* < 0.001
Peritoneum of the pouch of Douglas *	25.4 (18)	66.7 (60)	*p* = 0.002
Ovarian fossa *	29.6 (21)	38.9 (35)	*p* = 0.481
Retrocervical area *	38.0 (27) in parametrium and 5.6 (4) in the cervix	26.7 (24)	*p* = 0.391
Vesicouterine pouch *	-	25.6 (23)	
Endometrial implants on the ovaries *	35.2 (25)	8.9 (8)	*p* = 0.153
Posterior leaf of broad ligament #	18.3 (13)	2.2 (2)	*p* = 0.565
Serosa of the rectum	-	5.6 (5)	
Diaphragm	-	3.3 (3)	
Round ligaments	-	1.1 (1)	

* Pearson’s χ^2^ criterion; # χ^2^ test with Yates correction.

## Data Availability

The data presented in this study are available upon request from the corresponding author. The data are not publicly available because the database contains the personal data of patients.
